# Digital Twin for Training Bayesian Networks for Fault Diagnostics of Manufacturing Systems

**DOI:** 10.3390/s22041430

**Published:** 2022-02-13

**Authors:** Toyosi Ademujimi, Vittaldas Prabhu

**Affiliations:** Harold and Inge Marcus Department of Industrial and Manufacturing Engineering, Pennsylvania State University, University Park, PA 16802, USA; tta5@psu.edu

**Keywords:** smart manufacturing, Bayesian network, structure learning, digital twin, fault diagnostics, small data set

## Abstract

Smart manufacturing systems are being advocated to leverage technological advances that enable them to be more resilient to faults through rapid diagnosis for performance assurance. In this paper, we propose a co-simulation approach for engineering digital twins (DTs) that are used to train Bayesian Networks (BNs) for fault diagnostics at equipment and factory levels. Specifically, the co-simulation model is engineered by using cyber–physical system (CPS) consisting of networked sensors, high-fidelity simulation model of each equipment, and a detailed discrete-event simulation (DES) model of the factory. The proposed DT approach enables injection of faults in the virtual system, thereby alleviating the need for expensive factory-floor experimentation. It should be emphasized that this approach of injecting faults eliminates the need for obtaining balanced data that include faulty and normal factory operations. We propose a Structural Intervention Algorithm (SIA) in this paper to first detect all possible directed edges and then distinguish between a parent and an ancestor node of the BN. We engineered a DT research test-bed in our laboratory consisting of four industrial robots configured into an assembly cell where each robot has an industrial Internet-of-Things sensor that can monitor vibrations in two-axes. A detailed equipment-level simulator of these robots was integrated with a detailed DES model of the robotic assembly cell. The resulting DT was used to carry out interventions to learn a BN model structure for fault diagnostics. Laboratory experiments validated the efficacy of the proposed approach by accurately learning the BN structure, and in the experiments, the accuracy obtained by the proposed approach (measured using Structural Hamming Distance) was found to be significantly better than traditional methods. Furthermore, the BN structure learned was found to be robust to variations in parameters, such as mean time to failure (MTTF).

## 1. Introduction

With the increasing globalization of manufacturing, manufacturers are facing fiercer competition, leaving very little room for inefficiencies, such as downtime. In a recent survey of senior manufacturing professionals by Reference [1], 92% of respondents identified improving operational efficiency as their most significant business imperative. Fast and accurate fault diagnosis can significantly reduce downtime and is the most challenging phase of machine repairs [2,3]. 

Following the hierarchical nature of manufacturing systems, manufacturing faults can be classified as either machine-level (equipment) or factory-level (system) faults [4]. Machine-level faults pertain to the loss of functionality of individual components of a machine, such as the spindle bearing fault or axis servomotor fault, in a CNC machine. Errors caused by human operators running the equipment also fall under equipment-level faults. Factory-level faults, on the other hand, occur at the unit, cell, area, site, or enterprise levels in the integrated ISA-95 and ISA-98 model [5]. They refer to underperformance of the overall system or subsystem expressed as shortcomings in key performance indicators (KPIs), such as low overall equipment effectiveness (OEE), low production throughput, and high scrap rate. Examples of common manufacturing KPIs include those defined in the ISO 22400-1 [6] and ISO 22400-2 [7] standards. Because a manufacturing system consists of several machines, equipment-level faults are usually the root causes of factory-level faults. Despite this inherent relationship, system-level and machine-level fault diagnostics are usually modeled independently. However, a unified fault model of both fault classes can provide top management visibility on the effect of lower level faults on factory performance. 

The increasing adoption of smart manufacturing and Industry 4.0 is propelling the application of advanced data-analytics tools, such as machine learning (ML) models for improving performance, and additive manufacturing of functional parts [8]. Bayesian network (BN) is an ML algorithm that employs a graph-based representation to compactly encode the joint distribution of random variables in a single model, making it an ideal candidate for representing multilevel faults of complex hierarchical systems, such as manufacturing systems. Much of the existing diagnostics models are usually suited for single equipment rather than complex multilevel interconnected equipment [9,10]. BN’s compact representation allows for determining fault-propagation paths within and across fault classes, i.e., within factory-level faults and from factory-level fault to machine-level fault. Another significant advantage of BN is that a single model can be used for both diagnostics and prediction without the need for retraining, while modeling the associated uncertainties. It must be emphasized that, in contrast to most ML models that are black-box models, the graphical representation in BN allows for transparency, as the human expert can easily visually verify the model [11]. 

**Figure 1:**
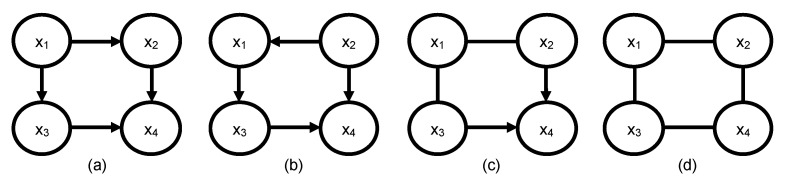
Illustration of independence equivalent graphs: (**a**,**b**) I-equivalent, (**c**) completed partially directed acyclic graph of (**a**,**b**), and (**d**) skeleton of (**a**,**b**).

The first step in training a BN model is determining the directed acyclic graph (DAG) structure. Common structure learning methods include either heuristic search methods that learn the DAG from observational data, eliciting the DAG from domain experts, or causal discovery through intervention on the real system. Heuristic search DAG learning algorithms require large amounts of complete balanced-class data to learn a DAG structure accurately. In contrast to other domains, such as image processing or social media, where available training data are in the hundreds-of-thousands to billions, manufacturing data are much smaller [12]. Furthermore, the amount of failure class examples in a dataset are usually much less than healthy class examples, creating a large class imbalance. Even with infinite balanced-class observational data, heuristic search algorithms cannot distinguish between graphs that are independently equivalent (I-equivalent) [13,14], such as graphs in Figure 1a,b. Incorrect edge directions in causal BN can result in misdiagnosis, which is undesirable. Thus, eliciting the whole or a subset of the DAG from domain experts is the most prevalent method. Expert opinion, however, may vary from one expert to the other, and there is no guarantee that an expert always remembers the influential relationship between variables. Although carrying out designed experiments is required for causal discovery, experimentation in a manufacturing environment will require shutting down normal production, and this can be prohibitively expensive.

In this paper, we propose the utilization of digital twin (DT) model to train BN that models both factory-level and equipment-level faults. A DT model can mirror the real operating conditions of a factory at both the system-level and machine-level, thereby simulating its real behavior [15,16,17,18]. Given that intervention is usually required for causal discovery [19] and the prohibitive cost of perturbing the physical production system for causal discovery, using a DT model is a more cost-effective alternative. 

Developing an all-purpose high-fidelity DT model for a complex manufacturing system is practically challenging, due to several reasons, including insufficient data and computational power [20]. DT models of multistage manufacturing systems are also uncommon in the literature, in comparison to single-stage and product DTs [21]. Therefore, we first propose a method to develop a multistage manufacturing system DT model specifically for multiclass fault diagnostics. Given that an ontology is usually required to develop an effective DT model for fault diagnostics but many organizations do not currently have an ontology [22,23], our proposed method utilizes natural language processing (NLP) data-tagging technique to mine the fault cause-and-effect relationships directly from maintenance log data. Additionally, considering the challenge of developing high-fidelity simulation models that mimic the behavior of a machine with fine granularity, we utilized co-simulation of original equipment manufacturer (OEM) simulator and discrete-event simulation (DES) model to actualize a DT model. The contributions of this research are as follows: (i) engineering of a detailed multilevel (equipment and system level) DT model, using co-simulation of equipment OEM simulator for process-level modeling and DES for system-level modeling showcased via an experimental robotic assembly cell; (ii) applying data-tagging NLP technique to extract fault events directly from qualitative data sources to facilitate high-fidelity fault modeling in a DT model; and (iii) utilization of a DT model for training of BN model for diagnostics of multilevel faults in manufacturing systems.

The organization of the rest of the paper is as follows. Firstly, an introduction to basic BN concepts is presented, including the current state-of-the-art in BN training and DT applications. Next, we discuss how the DT model was engineered, and its usage for training a BN is presented. We then present the experimental test bed setup that was used to validate the proposed approach before we conclude and discuss future research directions.

## 2. Background and Literature Review

A BN, denoted as a set (G,θ), graphically models the joint distribution of a set of random variables. G is a directed graph that represents the dependencies between variables, while θ represents the degree of influence between connected nodes in G. For fault diagnostics applications, these nodes correspond to faults, and a directed arc between two variables, such as $X_{j}\to X_{i}$, means that $X_{j}$ is the parent (or cause) of $X_{i}$, and $X_{i}$ is the child (or direct effect) of $X_{j}$, denoting a cause and effect relationship. Identifying the causal relationships among process variables is required for effective fault diagnostics [24], which is challenging to derive from observational data, as statistical dependency does not always imply causality [25]. Using the chain rule of probability, the joint probability distribution of a BN is given by the following:

(1) $$Pr\left(X\right)=\overset{r}{\underset{i=1}{\prod }}Pr(X_{i}|Pa\left(X_{i}\right))$$

where $Pa\left(X_{i}\right)$ is the parent set of node $X_{i}$, and r is the total number of nodes. 

### 2.1. Bayesian Network Structure Learning

BN structure learning methods can be generally classified as either heuristic search methods or other methods. Heuristic search methods learn the DAG from observational data and can be either score-based method, constraint-based method, or a hybrid of both methods. Other methods include expert opinion, using engineering models, and using design of experiment (DOE). 

Score-based methods search for a DAG that maximizes a goodness-of-fit score, and the search is a combinatorial optimization problem that is well-known to be NP-Hard [26]. Thus, a heuristic search is usually used in practice. The hill climbing (HC) algorithm is a popular score-based method that provides good tradeoff between computational demands and the quality of the models learned [27]. 

Constraint-based methods learn the structure from data by carrying out independence test to sequentially remove or add arcs. The undirected graph is determined first, followed by setting directions to v-structures (a node that has more than one parent) and finally directing the other arcs such that the acyclicity constraint is satisfied [28]. Examples of constraint-based algorithms are PC (Prototypical Constraint) [29], Grow–Shrink [30], and incremental association Markov blanket [31]. Finally, the hybrid method combines both score-based and constraint-based methods. 

Heuristic search structure learning methods require a large amount of balanced-class training data, but data are not always available in the required quantity, especially in manufacturing companies at the nascent stage of digitalization [32]. Large class imbalance is a result of the relative rarity of failure state in comparison to the healthy state. Even if adequate data are available, only the essential graph (skeleton and v-structures) can be learned at best from exploratory data, but the causal relationships between variables cannot be fully determined [13,33]. 

Expert opinion elicitation is the most preferred structure learning method in real-world applications, and it is either used to elicit the whole DAG or incorporated to improve DAGs learned using heuristic search methods, such as in References [34,35]. To obtain the DAG for diagnosing faults in a rolling manufacturing process, Li and Shi [24] integrated PC constraint-based learning algorithm with expert opinion. In Reference [36], the authors combined pairwise node-ordering knowledge elicited from an expert with a small observational dataset to determine the influential relationship between different human resource KPIs. Major challenges of expert elicitation include the misinterpretation of the BN edge direction due to variation between BN terminology and domain terminology [34]; inconsistency between experts; and experts’ memory-recollection limitations [36].

Engineering models developed by experts for other purposes embed some domain causal relationship knowledge and have been applied to determine BN structure. Examples include the failure mode and effect analysis (FMEA) model [37] and fault trees [38]. Cost models were utilized in Reference [39] to determine the BN graph for characterizing the influence of manufacturing decisions and variables on KPIs. A method to train BN using sensor data and maintenance log data for equipment-level fault diagnostics was proposed in Reference [40]. Carrying out designed experiments in the manufacturing plant is another BN structure learning method used in Reference [41]. Experimenting with a real manufacturing plant is, however, not economically feasible in most cases, and most of these approaches are limited to training BN for diagnosing either equipment-level or factory-level faults, but not both.

### 2.2. Digital Twin

The confluence of the maturity of computing technologies, Internet-of-Things (IoT) sensor technology, and faster Internet speed has given rise to the concept of cyber–physical systems (CPS). CPS is a technology that provides an interaction between systems via integrated communication, computing, and control [42,43], and this technology facilitates the integration of a physical asset with its virtual counterpart. A DT model is a virtual replica of a physical object connected via flow of information and data [44]. IoT sensors facilitate data collection from the physical system to update the DT model’s parameters in real time. Real time, here, can be hours, days, or even weeks, depending on the required decision-making timescale. This live connection between the physical and virtual worlds extends the DT’s use to timescales over which the physical object’s behavior will change significantly, thus preserving its representativeness throughout the physical object’s lifecycle [45]. A clear distinction between a digital model, digital shadow, and DT is that a digital model has no automated data connection to the physical system, while a digital shadow is a digital model with established unidirectional data connection from the physical equipment, and lastly a DT is created only when the communication is bidirectional [46].

DT models have been developed for improving the prognostic and health management of systems including fault diagnostics. A review of DT application in the maintenance domain is provided in Reference [47]. Physics-based models were utilized to develop a DT of a six-axis robot for predictive maintenance application in Reference [44], using a combination of OpenModelica (for creating the machine model) and MATLAB (for data processing). To extend the usage of a robotic cell’s design-phase digital model to production phase, automatic processing time updating was implemented via a code in the robot’s program in Reference [48] to obtain time stamps of the beginning and ending of a process. The up-to-date DT was used for improving production planning. A DT model for gearbox prognostics of a wind turbine was proposed in Reference [49]. In Reference [50], a DT model of a robot’s gripper with three failure modes was developed for remote monitoring, fault detection and diagnosis, and virtual commissioning. Processing variables were directly estimated from sensor data, and the DT model was used to detect anomalies in the gripper. The majority of these applications focus on single-equipment DT models.

A common DT modeling approach is to utilize an ontology model to describe the properties and relationships of the virtual model, such as in Reference [51], where a geometric ontology was used to create a DT model of a machined part. A reconfigurable assembly line DT model that uses ontology to describe the properties of the virtual layer’s five dimensions, namely geometric, rule, behavior, physical, and capability, was proposed in Reference [52]. Many organizations, however, do not have ontologies, as most ontology development approaches proposed in the literature have had very limited acceptability in industry [22,23]. We propose mining fault events and their effects directly from qualitative data sources, such as maintenance log data (or maintenance work order (MWO)), and corrective and preventive action (CAPA) report in this work.

Applications involving DT models of complex assembly shop-floor are less common in the literature because they are more challenging to construct than single-equipment DT models. In Reference [53], the authors developed a DT model for a satellite assembly shop-floor to fulfil smart production management and control. A multiscale (timescale and space scale) modeling method was proposed in Reference [54] for satellite assembly, integration, and test shop-floor modeling. For actualizing the DT model of a mine for maintenance optimization of multiple equipment, co-simulation of DES model and system dynamic cash-flow model was utilized in Reference [55] to study the influence of macroeconomic variables on the long-term time-based maintenance policy. A DES model was used to model the low-level interaction between equipment, while the cash-flow model was used to model high-level managerial profitability decisions. In this study, we also used co-simulation, where the equipment level was modeled using a simulator provided by the OEM, and the system level was modeled using DES, a commercial software.

BN models have been applied to create DT models, such as in Reference [56], where a non-parametric BN was used to model the health-state evolution process of the DT model for complex health-system monitoring. In Reference [57], a BN was used to model the decision system of the smart connection controller of a DT applied to telematics-based driving assistance. A dynamic BN was utilized to realize a DT in Reference [58] for aircraft-wing-health monitoring. To the best of the authors’ knowledge, despite the enormous amount of research focusing on DT technology for modeling manufacturing systems, a DT model is yet to be applied to train BN models for fault diagnostics. 

### 2.3. Data Extension

Data extension involves data augmentation/extension wherein an algorithm is used to generate additional data from the available small dataset or independent of it. Data generation has been effectively applied to improve ML model performance in cases with a small dataset [32]. Generative adversarial networks (GANs) are widely used generative models mostly in image-processing domain [59]. 

Other data-extension methods have also been proposed in the manufacturing domain, such as the particle swarm optimization (PSO) based virtual sample generation (VSG) method utilized to train forecasting models [60]. A VSG method based on Gaussian distribution was also used for training classification ML models in Reference [61], where the generalization ability of the classifiers on the combined synthetic and original training set outperformed that of the original training set only. The feasibility of using Kriging and Radial Basis Function models to generate data for learning BN model parameters was explored in Reference [32]. PSO was used to tune the parameter’s prior probabilities, and the authors reported that generated data could increase the accuracy of the trained networks. 

### 2.4. Digital Model for Data Generation

A different perspective from data extension is to generate data from a simulation model of the physical system. Given that running causal discovery experiments in real manufacturing systems for fault diagnostics could result in safety issues, lost production time, and damage to healthy equipment, leveraging their digital representation is a better alternative [62]. Simulation models embed the behavior of the real system and, thus, can be used to generate factory-like synthetic data for data analytics [16,17,63]. Data farming is an ongoing research area that focuses on generating data from simulation models and using data mining algorithms to uncover new knowledge from the generated data [64]. A general review of the use of DES model in conjunction with data analytics is presented in Reference [65]. 

In a small job shop where limited factory data are available, synthetic data were generated in Reference [66], using a virtual factory prototype to train an artificial neural network (ANN) model to predict the cycle time of incoming orders based on current shop-floor conditions. The virtual factory prototype was further used to generate data [16] for comparing the performance of ANN and Gaussian process regression models in predicting the cycle time of incoming orders. The ability to generate synthetic data from the virtual model allowed for the evaluation of different factory conditions, as well as the implementation of the model-selection step of ML training process before real data were available. To address the challenge posed by insufficient and imbalanced fault data in constructing prognostics models, Wang et al. [67] developed a DT model of an autoclave to generate fault data to train a convolutional neural network to enhance fault prediction.

## 3. Digital Twin Development

**Figure 2:**
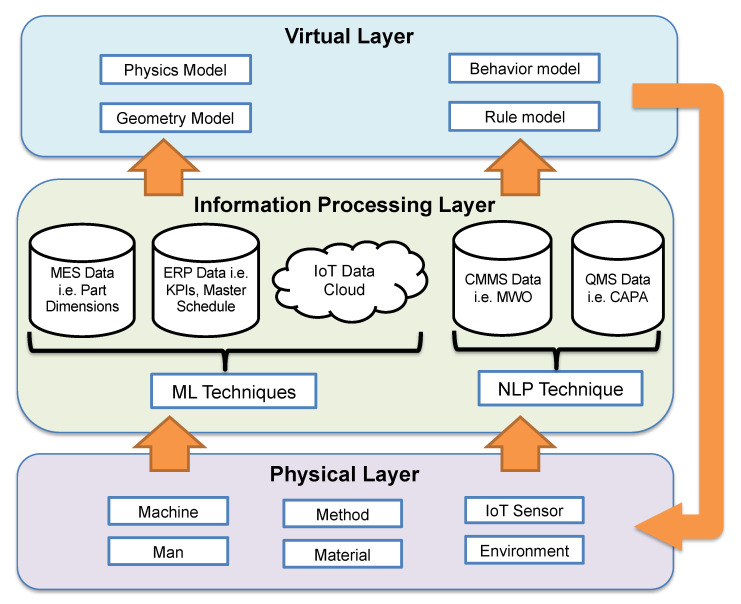
Three dimensions of the DT model.

We utilized a three-layer DT model framework consisting of a physical layer, virtual layer, and data/information-processing layer, as shown in Figure 2. The physical layer is the real physical system being modeled, while the virtual layer is a representation of the physical system in virtual space. The data/information-processing layer entails how data are processed and exchanged between the real system and virtual model. A DT model intended to train a BN model for both factory- and machine-level fault diagnostics application must include both factory-level and machine-level dynamics. That is, all the diagnosis data types collected in the manufacturing system must be included.

### 3.1. Physical Layer 

The physical space is a factory containing several production elements and production processes. Production elements refer to all entities that fall under the 4M1E, i.e., man, machine, material, method, and environment, while the production process includes the interaction between the production elements, such as part routings, equipment layout, and production logistics. The resolution of each element includes the fault modes and their effect on each element and the manufacturing system as a whole. 

### 3.2. Virtual Layer

The virtual layer is a virtual representation of the physical layer. It contains several models representing all the key elements and processes in the physical layer, including their system-level interaction as a manufacturing system. These virtual models are constructed in terms of their geometry, physics, behavior, and rule. The geometric and physical dimensions include the 3D CAD (computer aided design) model of the elements, as well as their physical properties, such as material, weight, and so on. The behavior dimension describes the operation dynamics logic of the element, while the rule defines its constraints, including association and deduction rules.

### 3.3. Data/Information-Processing Layer

This layer handles the continuous bidirectional data/information transfer between the physical layer and the virtual model, including the various data-processing techniques, to keep the virtual layer up-to-date with the physical twin. For the DT to support fault diagnostics at all levels, defect-occurrence events from quality management system (QMS) data, failure events from computerized maintenance management system (CMMS) data, and KPI data from enterprise resource planning (ERP) data, along with other IoT sensor data, must be fed into the DT model. For text data types, structuring of the raw data is required to obtain event data that can be modeled in the DT, i.e., failure modes and their effects.

#### 3.3.1. Qualitative Data Structuring Using Natural Language Processing

The raw text data, such as maintenance work order (MWO) from the CMMS database and CAPA report from the QMS database, being human-generated text written in natural language, oftentimes contain wrong spellings, abbreviations, and inconsistent terminology, making it impossible to automatically feed into the DT model. We are proposing utilizing the NLP algorithm proposed in Reference [68] to structure the data. The resulting structured data are in the form of cause-and-effect relationship, along with the frequency of occurrence of fault events, as well as repair time. The logic for each fault mode, as well as its effects, is modeled in the digital model. 

#### 3.3.2. Automatic Estimation of Processing Time, Batch Loading Time, and Part Travel Time

To automatically estimate the cycle time of a process, we utilized the software time-stamping approach used in Reference [48]. This cycle-time updating is implemented by adding some software code to the beginning and end of a machine’s program to trigger digital output signals when the program execution starts and ends. The time difference between these signals is then used as an estimate of the processing time. Because these signals are triggered in the equipment’s actual controller locally and only the results are exported, it does not suffer from signal latency when compared to sending a signal via TCP (transmission control protocol) to the edge computing device. Similarly, the batch loading time is estimated by setting a signal to trigger at the end and start of a batch, such that the time between signal activation is an estimation of the batch processing time (the longer duration) and batch loading time. Two separate signals could be used for this, as well, instead of a single signal. Lastly, setting up another digital output signal to track robot part drop-off time versus when the part reaches the next processing station (tracked by the proximity sensor) is a good estimate of part travel time between stations. This value can either be zero (meaning that there are already parts waiting to be processed) or can be some value which is the estimated travel time. This estimation works only when the time to travel from one station to the other is shorter than the time it takes to process a part. That way, the first part arrives at the subsequent station before the next part finishes processing.

#### 3.3.3. Automatic Downtime Estimation

During the lifecycle of a machine, the failure characteristics might change, as new faults not captured in historic data can occur. Therefore, to automatically update the equipment downtime, we propose using vibration sensor data in conjunction with proximity sensor data. Many industrial equipment, such as robots and CNC machines, vibrate when running. Based on this, the vibration data can be used to determine whether or not the machine is running via a classification or clustering ML algorithm. Because it is possible that the machine is not running because it was not scheduled to produce parts, the production schedule is factored into this estimation. Additionally, during normal production, to account for the times that the machine is waiting for parts to process, the proximity sensor data of incoming parts are used to estimate the starvation time of the machine. The equation for estimating the downtime is as follows:

(2) Downtime=TotalSensorDowntime−noProd−StarveTime

where TotalSensorDowntime is the time duration when the equipment is not running, estimated by using the vibration sensor data; noProd is the time duration when the machine was not scheduled to be producing; and StarveTime is the amount of time during normal production when the equipment is not producing because it is waiting for parts to process.

Although downtime information is also documented in maintenance log data and the failure parameters, i.e., mean time to repair (MTTR) and mean time to failure (MTTF), can be obtained from there, it is not uncommon to find errors in the date and time data [69,70]. Having two different data sources for failure time can help to improve the confidence in the failure time data in the case the estimates match, or allow for improving the data quality in the case where discrepancies exist. 

## 4. Bayesian Network Training Using Digital Twin

In this section, we propose methods to utilize the DT model developed in the previous section for training BN models for diagnostics of multilevel faults. 

### 4.1. Digital Twin Model for Structure Learning

Two methods are being proposed for DAG learning using DT. The first is using data-driven DAG learning algorithms to learn the DAG from synthetic data generated from the DT model, while the second method is to carryout intervention to learn the DAG in the DT model. 

#### 4.1.1. Data Generation Using Digital Twin for DAG Learning

The first proposed approach is to use the DT model to generate data and apply any BN heuristic search structure learning algorithm to learn the DAG from the generated data. As we illustrate using the experimental test bed in Section 5, this method suffers from the limitations of BN structure learning using observation data, which are only guaranteed to identify the skeleton of the of the DAG at best [71]. 

#### 4.1.2. Intervention Using Digital Twin for DAG Learning

The second approach entails applying structural intervention [14] to learn the DAG by using the DT model. Structural intervention involves forcing the value of the intervened variable to particular states and observing which other variables’ states change as a result of this intervention. We propose a Structural Intervention Algorithm (SIA) consisting of two steps: influence discovery step and parent-confirmation step. The pseudocode for SIA is presented in Algorithm 1 below. In the influence discovery step, a single variable, $y_{i}$, is intervened upon, and if this intervention changes the state of any other variable(s) $y_{j},\;\;\mathrm{for}\;j=1\;to\;n-1$, edge(s) are added between $y_{i}$ and each $y_{j},$ pointing in the direction of $y_{j}$, i.e., $y_{i}\to y_{j}$. Following this, if there are potential indirect causes that have edges between them, the parent-confirmation step is implemented by intervening on two variables simultaneously to distinguish between a direct cause (parent) and an indirect cause (ancestor).
**Algorithm 1** Structural Intervention AlgorithmGiven a variable set V with n variablesInitialize the edge set: E={ }
1.  Influence Discovery step:       for each $v_{i}$ in V
      intervene on $v_{i}$
        if the state of $v_{j}$ changes, where j≠i,           add edge $v_{i}\to v_{j}$ to E
     for each directed path $v_{l}\to v_{l+1}\to v_{l+2}\to \ldots \to v_{m}$ in E
        if there are any directed edges between $v_{l}\to v_{l+2},\;\ldots$
            Goto 22.  Parent Confirmation step:      Simultaneously intervene on $v_{l}$ and $v_{l+1}$ by fixing $v_{l+1}$ and perturbing $v_{l}$     if the state of $v_{l+2}$ does not change          remove edge $v_{l}\to v_{l+2}$return G=(V,E)


**Figure 3:**
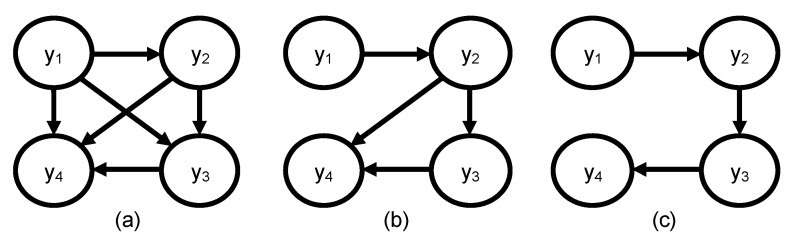
Example of resulting DAG using intervention. (**a**) After implementing influence discovering step. (**b**) After implementing parent-confirmation step for *y*_1_ and *y*_2_. (**c**) After implementing parent-confirmation step for *y*_2_ and *y*_3_, as this is the correct DAG.

To illustrate this, consider a BN whose correct DAG structure is shown in Figure 3c. If we assume that intervening on $y_{1}$ changes the states of $y_{2}$, $y_{3}$, and $y_{4}$, then intervening on $y_{2}$ also changes the state of both $y_{3}$ and $y_{4}$; and, lastly, intervening on $y_{3}$ changes the state of $y_{4}$, resulting in the DAG presented in Figure 3a. There is a possibility that the influence between $y_{1}$ and $y_{3}$ is through $y_{2}$, meaning that $y_{1}$ might be an ancestor of $y_{3}$. Upon carrying out the parent-confirmation step by simultaneously intervening on both $y_{1}$ and $y_{2}$, if the states of $y_{3}$ and $y_{4}$ do not change, then $y_{1}$ is conditionally independent of $y_{3}$ given $y_{2}$, and the resulting DAG is shown in Figure 3b. 

**Figure 4:**
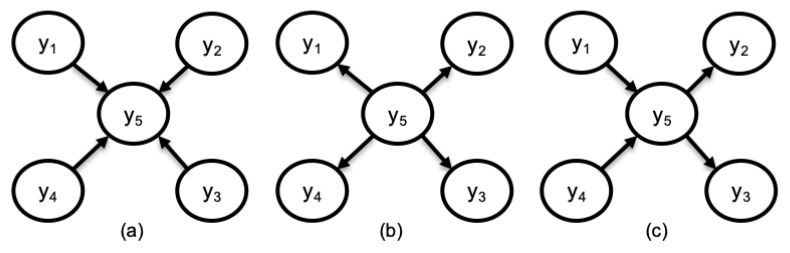
Example of common DAGs encountered in manufacturing domain used to compare pairwise search to SIA. (**a**) DAG representing multiple component assembly. (**b**) DAG representing distribution network. (**c**) DAG representing a combination of assembly and distribution.

The number of interventions required depends on the number of variables involved, the underlying structure of the network, and the sequence of implementing the interventions. This exhaustive search approach which would have been very expensive and time-consuming to carryout in a physical system, especially for BN with large number of variables, is much easier to implement in a DT model. The number of required interventions can also be reduced by incorporating other DAG learning methods, such as expert elicitation or data-driven learning methods, to limit the interventions to only edges with low confidence or some other criteria. Comparing SIA to pairwise DAG structure search where a single variable at a time is intervened upon and the resulting effect on another variable is observed, such as pairwise expert elicitation used in Reference [36], SIA requires fewer steps to discover the DAG. For example, using pairwise search, all the three DAGs in Figure 4a–c will require 20 steps to arrive at the correct DAG, while SIA will take 5, 5, and 7 steps respectively. It should be emphasized that, here, the SIA exploits the causal relationship between variables to implicitly prune the search space, as this can be a significant advantage in learning larger BNs.

### 4.2. Digital Twin Model for Parameter Learning

Following learning the DAG, any suitable parameter learning method, such as maximum likelihood estimation (MLE) or maximum a posteriori (MAP), can be used to fit parameters (conditional probability table (CPT)) to the DAG by using data generated from the DT model. DES models have the capability to generate data at specified sampling interval during the simulation run, e.g., every 1 or 2 or 5 s or minute, etc. This interval will depend on the frequency of occurrence that need to be captured in the data. Each instance (row) of the generated data will contain information about the current value/state of variables/events/objects of interest, e.g., whether or not a particular fault is currently active on a simulation object, the current value of KPIs, the current time, and so on.

## 5. Experimental Test Bed

To validate the proposed co-simulation and BN training approaches, an experimental robotic assembly line was set up in the laboratory. The assembly line consisted of four industrial ABB IRB 140 robots with IRC5 Single controllers arranged serially and was used to assemble 3D-printed interlocking plastic bricks. An industrial two-axis vibration sensor, incorporated with a temperature sensor, and with a sampling rate of 24 kHz programmed to sample every minute, was magnetically attached to axis two of each robot. The vibration data were uploaded directly to the cloud. The interlocking brick assembly consists of a base-brick onto which four other smaller bricks (top-bricks) are attached. Each robot attaches one top-brick onto the base-brick, making four total top-bricks per base-brick. To convey the parts from one robot station to the other, plastic U-channels inclined at an angle such that parts slide on freely were utilized and are here referred to as the slide rail. There are three U-channels in total. Proximity sensors wired directly into the digital input of the robot controllers were installed at the end of each U-channel to sense when an incoming part is available for pickup.

**Figure 5:**
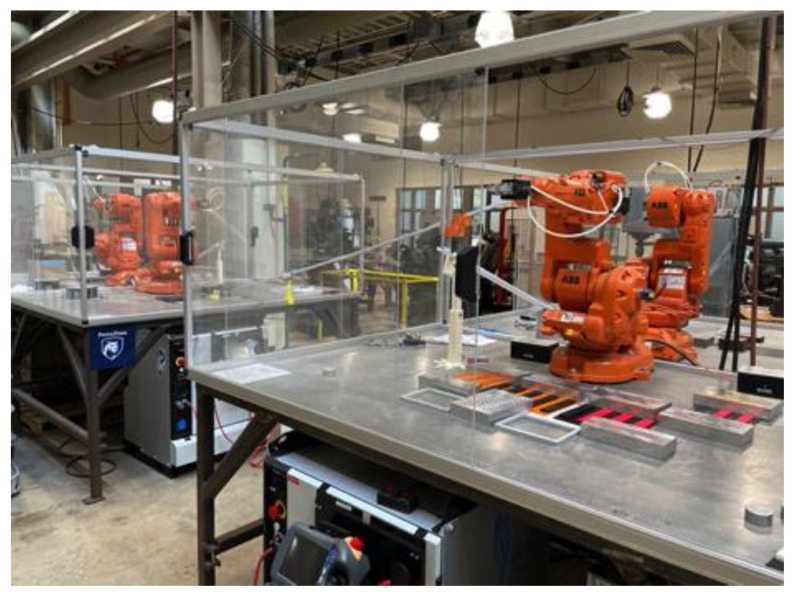
Physical experimental robotic assembly line setup showing the four robots.

**Figure 6:**
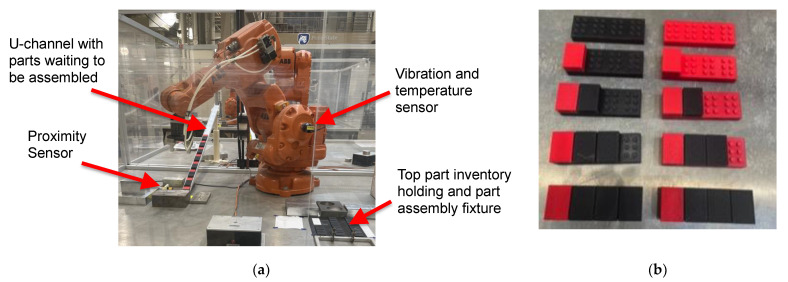
(**a**) Detailed view of a robot station. (**b**) Ten sample assembly parts, 5 on the left and 5 on the right, at various assembly states starting from empty assembly on top (base-brick only), followed by base-brick with one top-brick attached, base-brick with two top-bricks attached, and up to fully assembled at the bottom of the picture.

The experimental robotic assembly line is shown in Figure 5. The assembly process begins with a setup process of manually loading parts into fixtures positioned in each robot’s station. A batch of 20 top-bricks and 20 base-bricks are loaded in Robot Station 1 while 20 top-bricks each are loaded in the other robot stations. At the start of assembling, Robot 1 picks up a top-brick, places it on a base-brick, followed by picking the subassembly (base-brick with 1 top-brick attached) and placing it on the elevated end of the first U-channel. The partial assembly then slides down the U-channel under gravity to Robot 2 station. Once the subassembly arrives Robot Station 2, the proximity sensor is triggered, and this signals Robot 2 to go pick up the subassembly from the end of U-Channel 1. The subassembly is then placed in a fixture in Robot 2’s station, followed by the robot (Robot 2) fixing a top-brick to the appropriate location of the subassembly. Once assembled, the subassembly is sent to Robot Station 3, where a third top-brick is added before finally being sent to Robot Station 4. Robot 4 attaches the last (fourth) top-brick to complete the assembly process, and the fully assembled part is placed in a box. A detailed view of a robot showing attached sensors and samples of ten parts at different states of assembly are presented in Figure 6. The robot stations are reloaded with parts when they run out, and the assembly is run on a continuous basis.

The fault modes considered in this experiment are presented in Table 1. These few faults were selected based on the limitation that the robots are all healthy and cannot be damaged for the purpose of experimenting. They are relatively expensive equipment and are also used for other teaching and experimental purposes; thus, the faults are limited to those that can be readily simulated without causing actual damage to the robots. The parts being assembled are 3D-printed plastic parts, which are inexpensive, and intentionally defective parts can be readily fabricated. Quality inspection is carried out at each robot cell, and only correctly assembled parts are passed on to the next robot. The system-level performance of the robots was evaluated by their individual OEE metric, while the overall performance of the assembly line was measured by the production throughput (TH). The target TH is 70 parts per 2-h shift, and the processing time of one part is about 75.6 s on average.

### 5.1. Digital Twin Development

**Figure 7:**
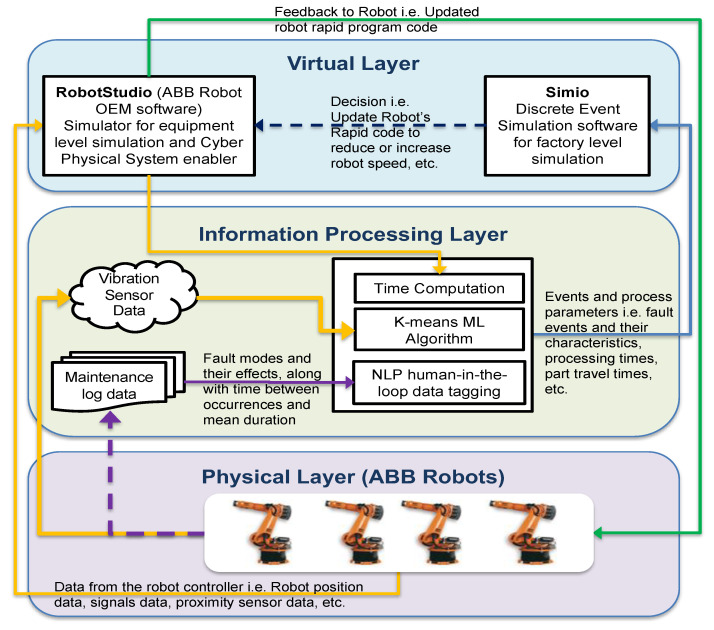
Schematic of co-simulation approach to actualize the DT model showing data-processing techniques used and communication between physical and virtual objects.

The DT model was implemented by using co-simulation of Simio DES software and RobotStudio simulator (ABB Robot OEM software). A schematic of the co-simulation approach with the data exchange and processing is shown in Figure 7. The unit-level model for each individual robot was created in RobotStudio, which interfaces directly with the physical robots’ controllers to form a cyber–physical system (CPS) and supports bidirectional data exchange. Data collected from the robot controller include robot tool-position data, proximity sensor data, and values of the digital output signals specified in the robot’s RAPID code. RobotStudio was also used for process-level offline simulations and generation of the robot’s RAPID program at the process-design stage, and also as a feedback mechanism for updating the robot’s program during production. RobotStudio simulator software is exactly the same as the software in the physical robot’s controller and, thus, can mimic its exact behavior. Sensor and qualitative data were collected during the run. The qualitative data were generated every time a fault event occurred by manually documenting the fault name, as well as its resulting effect. The system-level modeling, which involves the interaction between all robots as an assembly line, was modeled in Simio. All process-level data were used as input to the DES model in the form of events, their characteristics (i.e., failure events and their effects), and process parameter distribution estimates.

**Figure 8:**
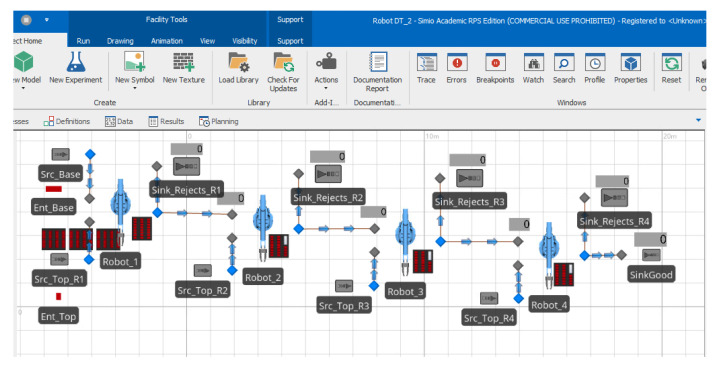
Two-dimensional view of the experimental robotic assembly line digital twin model in Simio.

A screenshot of the 2D view of the robotic assembly line DT model in Simio DES software is presented in Figure 8. Each robot is modeled using a Simio server object and has a Simio source object connected to it that supplies it top-bricks and a sink object where defective assemblies are routed. Robot 1 has an additional source for base bricks, while Robot 4 has an additional sink where fully assembled parts are routed to.

#### 5.1.1. Qualitative Data Analytics

Failure modes were extracted from the CAPA and MWO data, both of which are written in natural language. This manual documentation method by human operators usually introduces some errors, such as abbreviations, misspellings, and missing data. Moreover, the natural language format includes many irrelevant words and, thus, needs to be converted to a structured format. The NLP technique proposed in Reference [68] was utilized to structure the data to convert them to an easy-to-process cause-and-effect format. An example of two fault incidents entries is presented in Table 2, showing the raw format followed by the structured format. The NLP technique was first used to extract the keywords from the text and the misspellings corrected. Finally, a preferred label was added to identify common events that were documented using different words, such as the incidents in Table 2. The structured incidents were then modeled as events in the DT model, such as “controller_fault”, an event that causes the robot to be down. The downtime duration was also estimated from the data by subtracting the date and time recorded in the “Date Opened” field from that documented in the “Resolution Date” field. For log entries with missing dates, the downtime was estimated by using the vibration sensor data instead, such as for INC002 in Table 2. The MWO data are connected live to the DT, such that new events can be continuously updated in the DT.

#### 5.1.2. Automatic Estimation of Cycle Time, Part Restocking Time, and Part Travel Time 

The sensor data were used to automatically estimate cycle time, time to restock parts onto the robot fixture, the time for a part to travel from one robot cell to the other, and lastly the downtime of each machine. All of these estimations (except for the downtime estimation) were carried out by inserting code to activate a signal at specific locations in the robots’ RAPID program. RobotStudio software’s signal analyzer was used to collect the digital output signals used for these calculations. To implement the automatic process time update, a digital output signal of the robot was programmed to turn on for 1 s at the beginning and end of the robot RAPID program. The output data contain the value of the signal (0 or 1) and the corresponding time when the value was logged. The estimated processing time was obtained by subtracting the end time stamp from the beginning time stamp. This processing time was highly repeatable, because the robot programs always end and start in the home position and were more accurate (had less variance) than the values obtained by using a stopwatch. The part restocking time estimation was implemented by setting another digital output signal to turn on for 1 s at the end of the batch, as well as at the beginning of the batch. The time between this signal coming on (having a value of 1) is used as the estimate for the load time (the shorter time duration) and batch time (the longer time duration). These estimates are the natural parameter estimates, i.e., when the robots were healthy, and downtimes will inflate their values. 

Similarly, the part travel time between stations was estimated by setting a digital output signal to turn on for 1 s when a robot opens its jaw to drop a part on the U-channel rail. Provided that the U-channel is empty and has no parts on it, the difference between the time the U-channel’s proximity switch activates and the time the robot’s jaw closed is an estimate of the part travel time between robot stations. This estimation only works out when the U-channel is empty, i.e., when there are no parts queuing between stations. Moreover, this estimation is only possible because the distance between the stations are short, such that the time to process a part is much less than the time it takes to travel between stations. Whenever there is a queue between stations, the signal for the jaw-opening time stamp and the proximity sensor will both be active at the same time, and the difference will be zero. This provides a way of checking the validity of the estimate. 

#### 5.1.3. Automatic Estimation of Downtime 

An automatic estimation of the downtime from the vibration sensor data was implemented using K-means clustering, an unsupervised ML algorithm implemented in a semi-supervised manner. K-means partitions the data into k mutually exclusive clusters [72]. Euclidean distance was used as the clustering distance metric. The vibration sensor data were clustered into two groups to represent the high-vibration versus low-vibration states, where the low-vibration state corresponds to when the machine is off and the relatively high vibration corresponds to the machine-on state. The data cover a period when the robots are in a healthy state (no known underlying faults); thus, an increase in vibration, in this case, is only due to the robot being in use. The sensors used are industrial-grade accelerometer programmed to collect data every minute at 24 kHz sampling frequency. The per-minute sampling time is set by the sensor OEM and cannot be changed. This means that the resolution of the output time is 1 min, which is not a problem, as downtimes are usually in hours or days. 

**Figure 9:**
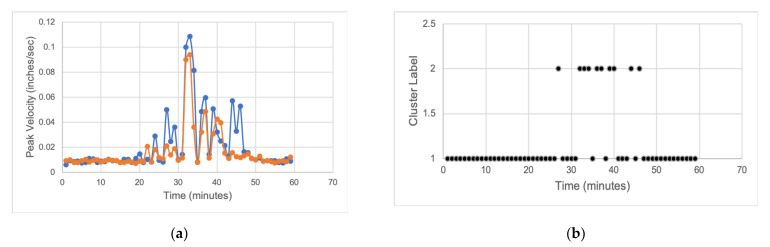
One hour of raw vibration sensor data and the resulting clusters after clustering for automatic estimation of downtime for a single robot: (**a**) *x*-axis and *y*-axis peak vibration velocity; (**b**) resulting clusters from applying clustering algorithm to the raw vibration data in (**a**), where clusters 1 and 2 correspond to low (off state) and high (on state) vibration states, respectively.

A simple illustration of automatic downtime estimation using an hour of vibration data is presented in Figure 9. A plot of the raw *x*-axis and *y*-axis peak vibration velocity data is shown in Figure 9a, while the clustering result of the raw data is shown in Figure 9b, where Clusters 1 and 2 correspond to low- and high-vibration states, respectively. From the graph, we can see that production began at the 28th min mark and ended at the 47th min mark, with some periods of starvation in between. Using the clustering data, the Total Sensor Downtime can be estimated as 50 min. An estimation of the starvation time from the proximity sensor data is 10 min. The total estimated downtime from using Equation (2) is 40 min. The recorded downtime using a stop watch was 39.4 min. When rounded up to the nearest minute, the results are the same.

An automatic update of downtime can be useful in some cases where the resolution date is missing in the maintenance log data, such as in INC002 in Table 2. The time stamps of the vibration data and the date maintenance log incident was opened can be matched to determine when the robot was back up running. In Figure 9, the zero time, which is the time the robot entered a downtime state, corresponds to the time INC002 was opened, which is 4:21 p.m. (see Date Opened row in Table 2). Using Figure 9, we see that the estimate of the missing resolution date is 28 min after the incident was opened, resulting in a resolution date of 4:49 p.m.

### 5.2. Digital Twin Validation

To ensure that the robotic assembly DT model accurately represents its physical twin, several verification and validation approaches were utilized. The equipment-level model using the RobotStudio ABB robot simulator contains the same software as the physical ABB robots, so the equipment-level dynamics are the same in a healthy robot state. We, thus, focus the validation on the factory-level model in Simio, which also models the robot faulty states. We start by visually verifying the simulation by watching it run to ensure the logic is implementing correctly. The model was then validated by comparing its output data to that of the physical assembly line. Because fault events occur randomly, OEE and cell part throughput data from 100 runs were compared to see if there is statistically significant difference between their means at a 95% confidence level. The null hypothesis is that the true difference in means is equal to zero for each KPI. From the two-sample t-test results presented in Table 3, the *p*-values are all greater than 0.05, meaning that there is no statistically significant difference between the means of the KPIs obtained from the DT model versus the physical assembly line. 

### 5.3. Bayesian Network Training

#### 5.3.1. Structure Learning

The first step in the BN training was to carry out an experiment on the real robot assembly line to determine the ground truth DAG, using the proposed SIA. The values of the two KPIs (OEE and TH) for the system-level faults were discretized according to the levels in Table 4, and the states of all equipment faults are binary, i.e., active or not active. Because fault nodes trigger upper-level faults, the experimentation began by activating each fault node and observing the values of the other variables in the model. For instance, activating the R1_POOS (Robot 1 parts out of stock) fault was implemented by removing top-bricks from the robot’s fixture, resulting in a defective assembly (the robot did not recognize that there were no top-bricks, so it kept on assembling defective parts). This, in turn, reduced the OEE value of Robot 1 (R1_OEE), due to the reduction in the number of good parts produced. Correspondingly, the states of Robot 2 OEE (R2_OEE), Robot 3 OEE (R3_OEE), Robot 4 OEE (R4_OEE), and TH also changed. 

**Figure 10:**
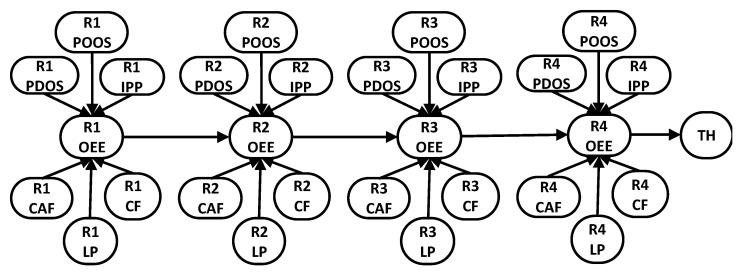
BN DAG learned from intervention on the physical experimental robotic assembly line, where R1, R2, R3, and R4 correspond to Robots 1, 2, 3, and 4 respectively. The fault acronyms are as presented in Table 1.

Once all of Robot 1’s faults were intervened upon, R1_OEE was intervened upon next. Because R1_OEE is a system-level fault, its value cannot be changed directly, but the underlying causes of quality, availability, or performance can be altered. Because of edges R1_POOS to R1_OEE, R1_POOS to R2_OEE, and R1_OEE to R2_OEE, a second parent-confirmation intervention step was required to verify if R1_POOS is a direct parent of R2_OEE. The second intervention was carried out by clamping the values of R1_POOS and R1_OEE at the same time, and observing if both variables still influence R2_OEE. Physically, a condition exists where R1_POOS can be made conditionally independent of R2_OEE. This condition can be implemented by feeding assembled parts to Robot 1, such that, despite being in a “parts out of stock” state, the output is still high. Viewing this from the Markov assumption perspective, if we observe the value of R1_OEE, we can predict the states of R2_OEE, meaning that observing R1_OEE blocks the path from R1_POOS to R2_OEE. Therefore, the path from R1_POOS to R2_OEE does not exist and is removed. After completing the intervention on all variables using the physical robot assembly line, the resulting DAG is presented in Figure 10. From the DAG, it can be seen that the effect of each robot fault is local (does not extend to other robots). Next, the intervention was carried out by using the DT model instead and the resulting DAG matched exactly the one obtained from intervention on the real physical robots, verifying our hypothesis that a DT model can be used to learn the correct DAG structure of a BN via experimentation.

To evaluate the performance of data-driven structure learning methods, 10,000 rows of sample data were generated from the DT model. The total sample size of data collected from the real system is about 100 rows of data, and this is very small. The BN models were created by using the bnlearn package [28] in R. Several data-driven learning algorithms were implemented, and their accuracy was compared by using structural hamming distance (SHD) metric, as shown in Table 5. SHD is a non-negative metric that counts the number of arcs that are different between the estimated DAG and the true DAG. Smaller values indicate a learned DAG with only a few incorrect edges, and a value of zero indicates that the learned DAG is the same as the true DAG. The Grow–Shrink constraint-based algorithm had the lowest SHD value of 13, and none of the methods was able to reproduce the correct DAG. This confirms the already-known limitation of heuristic search methods. 

**Figure 11:**
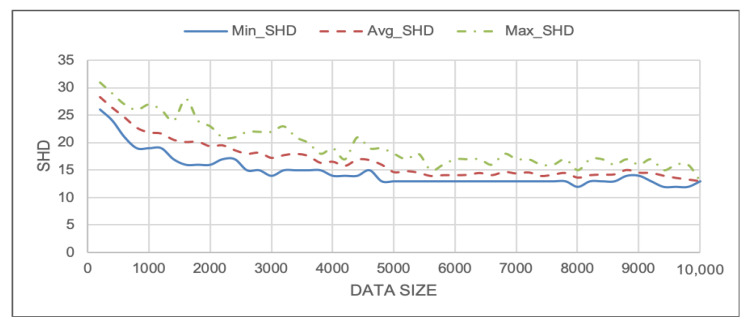
Effect of data size on the DAG obtained by using Grow–Shrink heuristic search BN learning method.

To evaluate the effect of sample size on the accuracy of the DAG, the data generated from the DT model were used to plot a data size versus SHD value. A subset of the whole data (a total of 10,000) was randomly sampled, starting with 200 rows and in increments of 200 until all the data were sampled. The Grow–Shrink algorithm was used in this analysis, because it performed the best out of all the data-driven algorithms tested. Because the subset was randomly sampled from the whole dataset, the resulting DAG will vary each time, depending on which particular data points were selected. Thus, 20 different samples were randomly drawn per data size, and the average, minimum, and maximum SHD values were computed. As it can be observed in Figure 11, the average SHD value reduces as the data size increases.

#### 5.3.2. Parameter Learning

**Figure 12:**
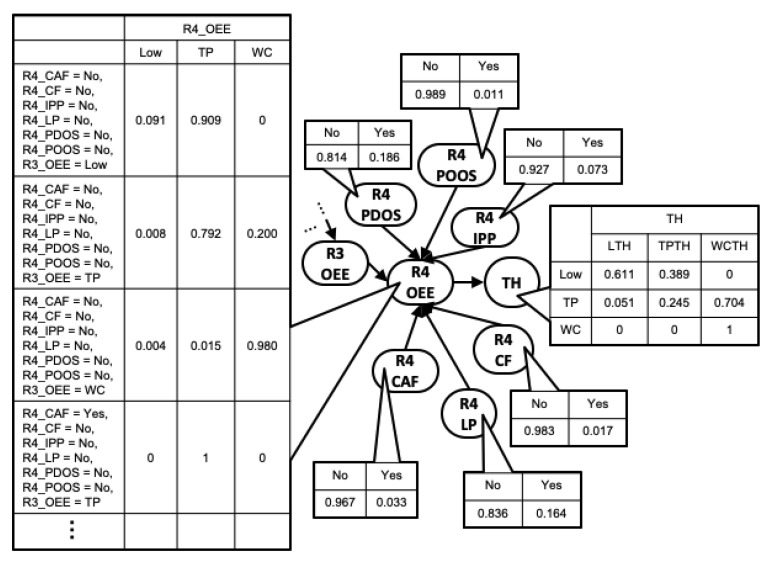
BN DAG and CPT showing Robot 4 OEE node, its parents, and child.

Lastly, MLE was used to fit parameters to the learned DAG. Due to size limitation, a partial view of the BN DAG structure and CPT is presented in Figure 12, showing only Robot 4 OEE node and its parents and child. Using the BN model, we can compute the probabilities of events conditioned on other events. For example, the probability of observing WC throughput, given that controller fault is active in Robot 4, is 0.605. The probability of the same event, given that controller fault is active in Robot 3 instead, is 0.695, and the probability increases to 0.744, given that the controller fault is active in Robot 1. This progressive increase in probability is a result of how the impact of upstream machine on a downstream machine can progressively reduce in a serial line. Likewise, the probability of observing typical OEE in Robot 4, given that R1_CAF is active—that is, $Pr(R4\_OEE=TP|R1_{CAF}=Yes$)—is 0.446, while the probability of the reverse (i.e., Pr(R1_CAF=Yes|R4_OEE=TP) is 0.066.

To evaluate the effect of the DT model’s accuracy on the BN model learned by using it, the DT model’s faults’ MTTF parameters were varied, and synthetic data were generated (see Table 6). Experiment 2 is the baseline model, and the other experiments involve reducing or increasing the exponentially distributed MTTF parameter. Ten thousand rows of data were generated for each experiment, and the data were used to train a BN model by using the Grow–Shrink heuristic search method. From the result, it can be seen that reducing the time to failure (Experiment 1) resulted in a better BN structure (lower SHD score) and increasing it resulted in a worse BN structure (Experiments 3 and 4). This is not surprising, as reducing the time to failure increases the fault occurrence frequency, thus increasing the balance of the data’s healthy class versus faulty class. However, learning the BN DAG by using our SIA is not affected by the failure frequency. In the case of the parameters learned, the Kullback–Leibler (KL) divergent score was used to evaluate the accuracy. A lower KL score is better, and a value of zero means that the parameters are the same as that of the baseline. From the KL result, changing the MTTF changes the learned parameters, but further studies are needed to qualify its sensitivity to the DT model’s parameters.

## 6. Discussion

A key distinction between DT and other advanced simulation models, such as digital shadows and information systems, is the DT’s automated bidirectional data exchange between the physical system and its twin [73]. The automatic DT parameter estimation approaches that were proposed are used to continuously update the DT model to keep the parameters up-to-date with its physical counterpart. New failure modes are also updated when they emerge by including their characteristics (i.e., effects, MTTF, and MTTR). Likewise, the simulator software can be used to update the physical robots’ program, thus completing the two-way information exchange requirement of a DT model. Continuous updating can also be extended to the BN model to keep it current by retraining it periodically to ensure up-to-date representativeness to maintain or improve its diagnostics accuracy. The automatic retraining of BN model using DT model will be part of future work. Although only non-invasive faults were considered in the experimental test-bed to avoid physically damaging the robots, the same modeling approach carries over to invasive faults as well. Implementing the DT model by using co-simulation of existing mature software potentially eliminates the time-consuming task of building one from scratch, and more importantly demonstrates the scalability of the proposed approach to industrial applications. Moreover, using the equipment OEM simulator ensures better representation of the physical twin in terms of overall behavior. Increasingly, OEMs are providing such equipment simulators which can be leveraged in DT modeling. A DT model engineered by using this approach will, however, be limited to the capabilities provided by the OEM simulator software. Because the scope of this research is fault diagnostics modeling rather than prognostics, we assume a binary health state of healthy and unhealthy, and we do not consider equipment degradation resulting from equipment wear and tear. 

Although BN has been used to develop DT models [56,57,58], we propose SIA to train BN by using the DT model in this work. The BN model structure (DAG) learned from carrying out intervention in the DT model is quite robust to deviations in the DT model parameters (i.e., deviations in fault frequency of occurrence), as it depends more on the flow of parts in the factory rather than on the values of the parameters. This is, however, not the case for data-driven heuristic search methods for BN structure learning. Deviations in the DT model parameters will have more effect on the BN model parameters learned and need to be investigated in the future. 

## 7. Conclusions and Future Work

Early research in digital twin (DT), which used to be called virtual system, studied real-time decision-making by using such approaches for production and maintenance scheduling [74,75,76,77]. In this paper, we explored a way to leverage DT to train a Bayesian network (BN), addressing key limitations of the current approaches in training BNs, including insufficient and imbalance-class data, the high cost of carrying out intervention on the factory floor, and the subjective nature of expert elicitation. Our proposed approach was validated by using an experimental assembly cell consisting of four industrial robots that were engineered using co-simulation of original equipment manufacturer simulator and discrete-event simulation model. The BN structure obtained via carrying out an intervention in the DT model was identical to that obtained from intervening on the experimental assembly cell, thus validating the use of DT model for carrying out intervention for BN structure learning in this instance. The Structural Intervention Algorithm proposed in this paper uses an Influence Discovery step to first detect all possible directed edges, followed by a Parent Confirmation step to distinguish between a parent and an ancestor. It should be emphasized that, here, the SIA exploits the causal relationship between variables to implicitly prune the search space, which can be a significant advantage in learning large BNs.

In regard to future avenues of research—being that the proposed approach was validated using an experimental robotic assembly line—implementing the DT model in real manufacturing plant is a next step. Moreover, the application of small dataset parameter learning methods, such as qualitative maximum a posteriori (QMAP) to BN models containing both factory-level and equipment-level manufacturing fault types, need to be studied. The sensitivity of the learned BN parameters to deviations in the DT model parameters also needs to be evaluated. Furthermore, the effect of discretization levels on the BN structure learning algorithm should be pursued. Another fruitful area of future work would be to explore the computational tractability of the SIA algorithms for large BNs, along with techniques to prune search space in the Parent Confirmation step. Lastly, given that more data can sometimes hurt the model and a large amount of data can be generated from a DT model, identifying a method to determine the optimal amount of synthetic data samples to generate for maximum performance of the structure learning and parameter learning algorithms is desired.

## Figures and Tables

**Table 1:** Robotic assembly-line faults.

**Part Defect Type Quality**	**Category**	**Failure Implementation**
Part (Top-Brick) Dimension Out of Specification (PDOS)	Quality Fault	Print top-brick with out-of-specification dimensions
Incorrect Part (Top-Brick) Placement (IPP)	Quality Fault	Place top-brick such that it does not completely fit inside fixture
Parts (top-brick) out of Stock (POOS)	Quality Fault	Remove top-brick from the part fixture
**Machine Failure Event**	**Category**	**Failure Implementation**
Compressed Air Fault (CAF)	Machine Fault	Turn off the compressed air supply switch to the robot
Controller Fault (CF)	Machine Fault	Stop the robot program
Low Performance (LP)	Machine Fault	Reduce the speed of the robot in the program

**Table 2:** Example of two maintenance log failure incidents in both raw and structured formats.

Input Field	Raw Data	Structured Data	Raw Data	Structured Data
Incident ID	INC001	INC001	INC002	INC002
Asset ID	Rob001	Rob001	Rob001	Rob001
Equipment Name	Robot 1	Robot 1	Robot 1	Robot 1
Date Opened	31 March 2021 10:41AM	31 March 2021 10:41AM	1 April 2021 04:21PM	1 April 2021 04:21PM
Problem Description	The robot is not turnin on.	robot_is_down	Robot will not switch on	robot_is_down
Resolution Status	Closed	Closed	Closed	Closed
Resolution Notes	Robot controller is fualty.	controller_fault	Controller is not booting.	controller_fault
Resolution Date	31 March 2021 11:41AM	31 March 2021 11:41AM		

**Table 3:** Two-sample *t*-test results at 95% confidence level for difference in means of KPIs obtained from digital twin model and physical twin for model validation.

KPI	Mean for Digital Twin Model	Mean for Physical Assembly Line	*p*-Value
Robot 1 OEE	0.9441	0.9345	0.9206
Robot 2 OEE	0.9112	0.9123	0.9247
Robot 3 OEE	0.9115	0.9110	0.9992
Robot 4 OEE	0.9087	0.9099	0.8736
Cell Throughput	101.171	101.209	0.8769

**Table 4:** Throughput and OEE KPIs discretization levels.

KPI	Low (L)	Typical (TP)	World Class (WC)
OEE	Low≤0.4	0.4<TP<0.85	WC≥0.85
Throughput (TH)	LTH≤60	60<TPTH<80	WCTH≥80

**Table 5:** Comparison of heuristic search structure learning algorithms, using SHD value. Lower SHD value is better.

	Score-Based Algorithm		Constraint-Based Algorithms	
Data	Hill Climb	Tabu Search	Grow–Shrink	PC
Small dataset from physical line (100 Rows)	45	45	23	23
Data generated from digital twin (10,000 rows)	55	55	13	21

**Table 6:** Evaluation of the effect of DT model accuracy on the resulting structure learned by using Grow–Shrink heuristic structure learning method and parameters learned by using MLE.

Experiment	Mean of the Exponentially Distributed Time-to-Failure for Each Fault (Hours)						SHD Score	KL Score
	CAF	CF	PDOS	IPP	POOS	LP		
1	25	50	3.5	12	20	3	9	173.47
2 (baseline)	50	100	7	24	40	6	13	baseline
3	100	200	14	48	80	12	16	292.07
4	200	400	28	96	120	24	21	193.47

## Data Availability

Not applicable.
